# Permeabilizing Cell Membranes with Electric Fields

**DOI:** 10.3390/cancers13092283

**Published:** 2021-05-10

**Authors:** Alondra A. Aguilar, Michelle C. Ho, Edwin Chang, Kristen W. Carlson, Arutselvan Natarajan, Tal Marciano, Ze’ev Bomzon, Chirag B. Patel

**Affiliations:** 1 Molecular Imaging Program at Stanford, Department of Radiology, Stanford University School of Medicine, Stanford, CA 94305, USA; aaa2334@columbia.edu (A.A.A.); michellechelseaho@berkeley.edu (M.C.H.); echangcv@stanford.edu (E.C.); anataraj@stanford.edu (A.N.); 2 Beth Israel Deaconess Medical Center, Department of Neurosurgery, Harvard Medical School, Boston, MA 02215, USA; kwcarlso@bidmc.harvard.edu; 3 Novocure, Ltd., 31905 Haifa, Israel; TMarciano@novocure.com (T.M.); ZBomzon@novocure.com (Z.B.); 4 Department of Neurology & Neurological Sciences, Division of Neuro-Oncology, Stanford University School of Medicine, Stanford, CA 94305, USA

**Keywords:** alternating electric fields (AEFs), bioelectrorheology, cancer, cell membrane, cell modeling, electroporation, glioblastoma, tumor treating fields (TTFields), voltage-gated ion channel

## Abstract

**Simple Summary:**

The FDA recently approved a fourth approach (in addition to surgery, radiation therapy, and chemotherapy) for treating glioblastoma; namely, tumor treating fields (TTFields), a form of alternating electric fields (AEF) therapy that is delivered to the tumor via electrodes placed on the scalp. Despite prolonging overall survival by 5 months when combined with standard chemotherapy in patients with newly diagnosed glioblastoma, the mechanisms of action of TTFields are not fully understood and primarily involve its interruption of mitotic spindle formation which impairs cancer cell division. A novel mechanism of action of TTFields at the cell membrane was recently identified, in which TTFields increases cancer cell membrane permeability. This finding could be exploited to enhance drug delivery to cancer cells. Here, we review the likely mechanisms by which TTFields permeabilize cancer cell membranes, i.e., voltage-gated ion channels, bioelectrorheological effects, and electroporation. Finally, we discuss an explanatory formulation that incorporates all three models.

**Abstract:**

The biological impact of exogenous, alternating electric fields (AEFs) and direct-current electric fields has a long history of study, ranging from effects on embryonic development to influences on wound healing. In this article, we focus on the application of electric fields for the treatment of cancers. In particular, we outline the clinical impact of tumor treating fields (TTFields), a form of AEFs, on the treatment of cancers such as glioblastoma and mesothelioma. We provide an overview of the standard mechanism of action of TTFields, namely, the capability for AEFs (e.g., TTFields) to disrupt the formation and segregation of the mitotic spindle in actively dividing cells. Though this standard mechanism explains a large part of TTFields’ action, it is by no means complete. The standard theory does not account for exogenously applied AEFs’ influence directly upon DNA nor upon their capacity to alter the functionality and permeability of cancer cell membranes. This review summarizes the current literature to provide a more comprehensive understanding of AEFs’ actions on cell membranes. It gives an overview of three mechanistic models that may explain the more recent observations into AEFs’ effects: the voltage-gated ion channel, bioelectrorheological, and electroporation models. Inconsistencies were noted in both effective frequency range and field strength between TTFields versus all three proposed models. We addressed these discrepancies through theoretical investigations into the inhomogeneities of electric fields on cellular membranes as a function of disease state, external microenvironment, and tissue or cellular organization. Lastly, future experimental strategies to validate these findings are outlined. Clinical benefits are inevitably forthcoming.

## 1. Introduction

**Figure 1:**
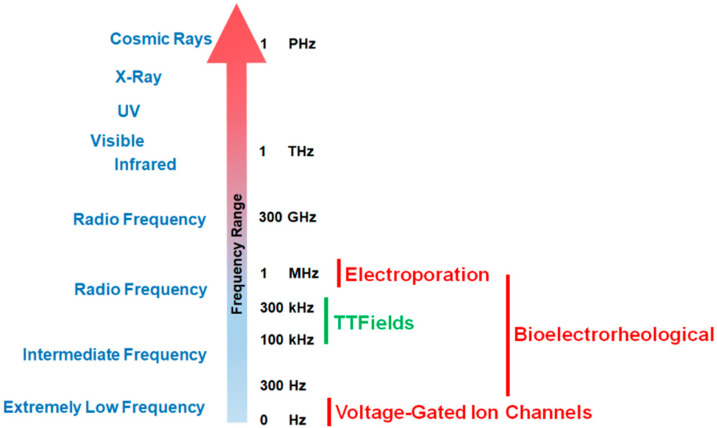
Effective working frequency ranges of voltage-gated calcium channels, tumor treating fields (TTFields), the bioelectrorheological model, and the electroporation model along the electromagnetic spectrum. As shown in the figure, TTFields falls within the range of intermediate frequencies while calcium channels operate at very low frequencies. By way of contrast, electroporation usually operates within the radio frequency ranges (television, radio, cell phones, microwave) while the bioelectrorheological model spans intermediate to radio frequencies.

Since the phenomenon’s initial discovery, the impact of exogenous electric forces on biology has prompted numerous research investigations. An iconic example is the experiment by Luigi Galvani in which electricity from lightning storms or static generators induced the twitching of the legs from frogs [1,2]. Advances in techniques to measure voltage and current gradients in biological material coupled with imaging technology to assess morphological changes in organisms have revealed that exposure to electro-magnetic occurrences can trigger a range of morphometric processes from embryological development to wound healing [2,3,4]. Frequencies for biologically relevant exposure to electromotive forces (EMFs) can range from being very low (0–300 Hz) to intermediate values (30 Hz to 400 kHz) to high and very high frequencies (1 MHz to 10 GHz, see Figure 1 [2,5]).

Different EMF ranges will initiate different biological effects (Figure 1). For example, low frequencies (0–300 Hz) tend to trigger membrane depolarization and consequently stimulate nerve, muscle, heart, and other tissues [2]. At the other end of the spectrum, high frequency electric fields (120 MHz) were found to result in a reversible elongation accompanied by a rotatory motion of the cells due to stress resulting from field distortions [6]. The process of electroporation (i.e., microbiology technique in which one employs a pulse of electricity to briefly open the pores in the cell membranes primarily for the purposes of DNA transduction) occupies a wide frequency range from 1 Hz to 1 MHz [7]. Intermediate frequency (100–400 kHz) alternating electric fields (AEFs), referred to as tumor treating fields (TTFields) in the context of cancer, have been studied in detail for many years [8,9,10]. These electric fields have been applied to glioblastoma, the most common and lethal form of primary brain cancer in adults [10,11]. Based on the results of a phase III clinical trial that showed 200 kHz TTFields in combination with adjuvant temozolomide chemotherapy prolonged the median overall survival (OS) in newly diagnosed GBM patients by 4.9 months (and prolonged median progression-free survival [PFS] by 2.7 months) compared to adjuvant temozolomide alone, this form of AEF therapy was approved by the U.S. Food and Drug Administration (FDA) in 2015 [11,12]. The OS benefit due to the addition of 200 kHz TTFields therapy was maintained regardless of glioblastoma patient subgroup, i.e., MGMT promoter methylation status, extent of resection (biopsy, partial, gross total), region (U.S. vs. non-U.S.), age (<65 vs. ≥65 years), Karnofsky performance score (90–100 vs. ≤80), and sex [12]. Of note, the 5-year OS rate increased from 5% to 13% in the glioblastoma patient arm receiving 200 kHz TTFields [12]. More recently, in 2019, 150 kHz TTFields received FDA approval under its humanitarian device exemption for the first-line treatment of unresectable, locally advanced or metastatic, malignant pleural mesothelioma (MPM) when combined with pemetrexed and platinum-based chemotherapy [13]. In this single-arm phase II clinical trial, the median OS and PFS were 18.2 months and 7.6 months, respectively [13], compared to corresponding historical controls of 12.1 months and 5.7 months from a 2003 phase III clinical trial evaluating pemetrexed in combination with cisplatin in MPM [14]. Of note, a randomized controlled open-label phase III clinical trial in MPM patients published in 2016 demonstrated a median OS in its control arm (pemetrexed and cisplatin) of 16.1 months [15]. The treatment of cancers by TTFields is thus a novel, validated therapy that may represent an additional paradigm (alongside surgery, radiation therapy, chemotherapy, and immunotherapy [16]) in anti-cancer treatments [17].

**Figure 2:**
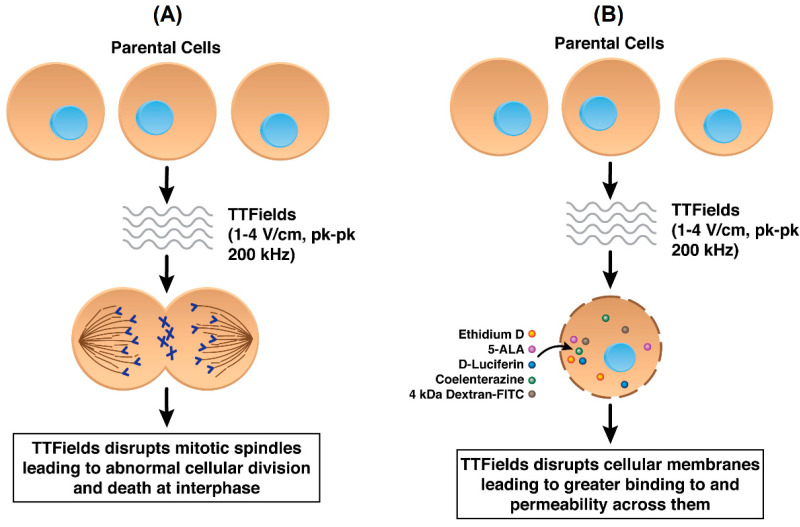
(**A**) Standard mechanism of action of TTFields in cancer cells by disrupting mitotic spindle formation (**B**) TTFields disrupting cancer cell plasma membranes resulting in increased permeability.

While not yet fully ascertained, the mechanisms of anti-cancer action by TTFields include their destabilizing effect on the tubulin dimers that have intrinsic dipole moments and which are the building blocks of microtubules, which in turn constitute the mitotic spindle [8]. By forcing microtubular filaments to align along electric field lines through the exogenous imposition of TTFields, the functionality of the mitotic spindle is interrupted in actively dividing cells [18] thereby disrupting replication [8,9,19] (Figure 2A). Such perturbations lead to abnormal chromosomal segregation, mitotic cell death, and perhaps apoptosis from cells that are able to exit mitosis [8,9,19]. Numerous proof-of-concept experiments as well as relevant technological developments have occurred over the last ten years [8,19], culminating in the approval by the FDA of a commercial, clinical TTFields device (Optune^®^, Novocure Ltd., Jersey, UK) for the treatment of newly diagnosed and recurrent glioblastoma [11,12,19,20,21,22,23].

However, it is widely believed that AEFs in general, and TTFields in particular, display a wider range of mechanisms. In addition to destabilizing microtubules, AEFs may affect the formation of proteins involved with the mitotic spindle complex, e.g., septins [24,25]. Investigations have been made to examine the role of AEFs in disrupting DNA replication, inducing autophagy, and impacting immune cell viability and function [5]. Recently we have demonstrated that TTFields exposure, in conjunction with a novel anti-cancer compound Withaferin A, synergistically inhibited the growth of human glioblastoma cells [26]. We hypothesized that such a synergistic effect is due to increased accessibility of Withaferin A to glioblastoma cells through TTFields’ capability to transiently increase tumor cell membrane permeability (Figure 2B). As shown by scanning electron microscopy (SEM), TTFields induce fenestrae in cancer cellular membranes through increases in the size and number of observed membrane openings post-exposure [27]. This latter finding suggests cancer cells have intrinsic physico-chemical properties that differ from cognate non-cancer cells and thus may explain their differential responsiveness to AEFs. Indeed, cancer cells are known to possess a relatively more depolarized resting membrane potential compared to non-cancer cells [28,29,30,31]. Cancer cells are more deformable compared to non-cancer cells because of their altered membrane composition; consequently, cancer cells’ responsiveness to fluid shear stress has been reported to be different from that of non-cancer cells [32,33,34]. There are also alterations in ion channel expression, membrane distribution, and function in cancer cells, which indicates how ion channel changes could serve as biomarkers of tumor progression [35]. As described in subsequent sections in this review, the aforementioned properties of cancer cells can be impacted by AEFs.

Such properties also have implications for cancer therapeutics. Such effects were shown to occur in cancer cells but not in non-cancer cells [27]. Additive, and maybe synergistic, optimization in efficacy of combining chemotherapies with TTFields can be uncovered by understanding how parameters such as frequency, field strength, and duration of TTFields exposure optimize shape and number of fenestrae in cancer cell membranes. In order to understand such optimization, it is crucial for us to produce workable models of how TTFields (or AEFs in general) interact with cellular membranes.

In theoretical studies (by authors TM and ZB, personal correspondence), analytical models demonstrate that the low-electric field intensity (1–3 V/cm) delivered by TTFields to single rounded cells cannot generate significant physical effects (e.g., dielectrophoretic force, electrostatic pressure, dipole orientation, or heat) in the cytoplasm. Previous simulation studies [36] have shown that amplification of the applied signal could be generated at the cleavage furrow during cell division. Indeed, cellular and pre-clinical studies have demonstrated that dividing cells are affected by TTFields during mitosis [8,37]. Instead, we demonstrate that important amplification of the signal at TTFields frequencies could occur at the membrane pore or channel level, or in the intercellular spaces between confluent cells. This amplification, therefore, mainly occurs in the vicinity of the cell membrane, where most of TTFields’ physical effects are expected to occur.

**Figure 3:**
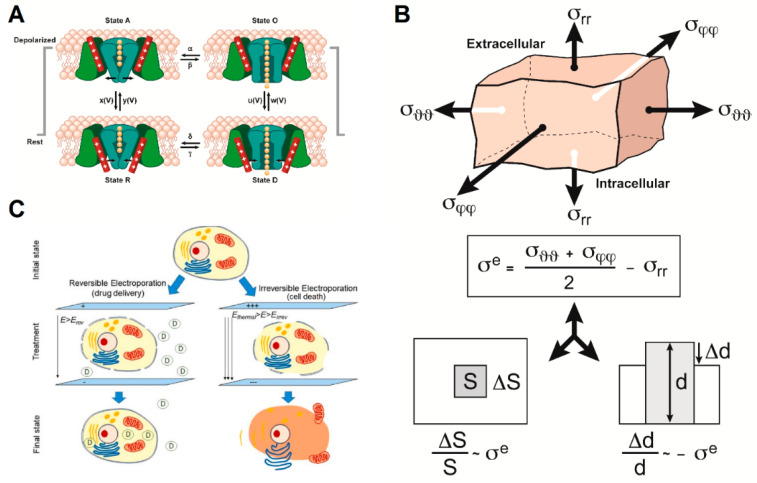
The three models that could partially explain the action of alternating electric fields (AEFs) on cell membrane integrity and function include: (**A**) Impact of AEFs on voltage-gated ion channels (adapted from [38,39]). (**B**) The bioelectrorheological model (adapted from [40]). (**C**) The electroporation model (reprinted with permission from ref. [41]. Copyright 2019 Springer Nature). Parameters are defined in the respective references and Tables S1–S3.

We propose three models that explain how intermediate frequency AEFs affect cellular membranes (Figure 3) and impact membrane permeability. First, AEFs may affect cellular membrane permeability by imposing an EMF gradient that forces voltage-gated ion channels to adopt an “open” state and thus cause increased ionic and molecular permeability (Figure 3A and Table S1). Second, alternating electric fields of 1 kHz to 1 MHz can theoretically impose shear stress on membranes, leading to membrane distortions and potential changes in permeability (the “bioelectrorheological model”, as shown in Figure 3B and Table S2). Third, AEFs, and TTFields in particular, share many characteristics with reversible electroporation (Figure 3C and Table S3). In the following sections, we systematically describe the features of each model and outline how selected elements from each can be part of a synthesis that best describes the impact of AEFs and TTFields on cancer cell membranes.

## 2. Results: Explanatory Models

### 2.1. Ion Channel Activation through Effects of AEFs

The effects of alternating current (AC) electric fields on ion channels have been investigated extensively in the ranges of frequency and amplitude that affect nerve cells (neurons and nerve fibers), e.g., from direct current (DC) to 50 kHz and from 0.001 V to 1 V along the fiber, and to a lesser extent, at other frequency ranges and amplitudes [42]. Prima facie, while the amplitude of TTFields is sufficient to modulate ion channels in the cell membrane, the frequency of TTFields (100–300 kHz) would seem to preclude effects such as initiation and propagation or blocking of action potentials in nerve cells, because such effects are not seen above ~30–50 kHz [2]. Above this frequency range, the time constants of the channel gates, being 1–2 orders of magnitude greater than the periods corresponding to TTFields’ frequencies, are too long for the gates to track the amplitude modulation of the sine wave. The membrane capacitance filter becomes negligible as well, consequently resulting in the inability of a nerve fiber to initiate and propagate an action potential (see Table S1) [42,43,44,45].

Yet, there is also empirical evidence demonstrating that TTFields stimulation does cause effects on the ion channels of cellular membranes. For example, using several empirical techniques, Neuhaus et al. [46] showed that 200 kHz AEFs at 1 V/cm (i.e., TTFields) affected a voltage-gated calcium channel (Ca_v_1.2) in T98 human glioblastoma cells and produced apoptosis as well as G1 or S phase cell cycle arrest, breakdown of the inner mitochondrial membrane potential, and DNA degradation [46]. They noted that anti-clonogenicity was significant but low at 10% after 5–7 days [46]. The low efficacy is probably due to the lower field strength (1 V/cm in Neuhaus et al. [46]) compared to what Kirson et al. [47] showed is needed for higher efficacy: 2–3 V/cm [47]. The latter setting resulted in a reduction in cancer cell number [47]. Neuhaus et al. also did not change the AEF direction with respect to the cells, which significantly increases efficacy as demonstrated by Kirson et al. [47]. These two factors may also be responsible for the lack of similar effects on Ca_v_1.2 channels in U251 human GBM cells studied by Neuhaus et al. [46]. In summary, Neuhaus et al. showed correlation of AEF effects on Ca_v_1.2 channels with previously reported TTFields’ effects, but not causation, probably due to low AEF field strength and use of a single-direction electric field [46].

Two key questions arise: 1. Through what mechanism do TTFields modulate voltage-gated calcium channels (Ca_v_^2+^)? and 2. Is the effect epiphenomenal or integral to TTFields’ mechanism of action? The first question is of interest to the general theory of how electromagnetic fields affect cells and tissues, and the second, to understanding how TTFields kill cancer cells, thereby improving TTFields’ efficacy.

To answer the first question, two theories have been proposed for how electromagnetic fields modulate Ca_v_^2+^ channels: (1) by depolarization of the cell membrane, which is typically employed in mechanism of action explanations of voltage-gated ion channels such as Na_v_^+^, K_v_^+^, and Ca_v_^2+^ [48] and (2) by direct action on the charged residues in the voltage-sensing S4 segment adjacent to the pore that opens and closes the channel [49,50]. The second theory provides an explanation for effects on ion channels at frequencies above those at which neurons and nerve fibers respond.

A second piece of evidence supporting TTFields’ effects on cell membranes comes from finite element modeling in which a modified Schwan equation was the governing model for membrane depolarization (along with the Laplace equation for electric field distribution). Li et al. [51] predicted that TTFields depolarize the cell membrane by 10-17%, which may be enough to open membrane Ca_v_^2+^ channels [51] and, at TTFields’ frequency, would likely ‘freeze’ the channels in an open state.

However, if we assume TTFields act on voltage-gated Ca_v_^2+^ channels, the diameter of the channels is on the order of a few nanometers and therefore is too small to associate them with the pores shown by Chang et al. to be created or enlarged by TTFields, which had cross-sectional areas of 240.6 ± 91.7 nm^2^ [27], representing diameters of 17.5 ± 10.8 nm. For this reason, the remainder of this review focuses on the bioelectrorheological and electroporation models.

Further, we conclude that even if TTFields open Ca_v_^2+^ channels, the effect would be secondary to the effect of the pores found by Chang et al. [27]. The size of the pores compared to ion channel diameters and their presumed non-ion-specificity implies that, by the Nernst and Goldman equations [52], complete equalization of all ion differentials across the cell membrane would occur rapidly and fully depolarize the cell. While this hypothesized depolarization should also trigger Ca_v_^2+^ channel opening, any effects from Ca^2+^ conductance would be superfluous to those via the larger pores.

### 2.2. Bioelectrorheological Model

The bioelectrorheological model of the cell was proposed and has been expanded upon through a series of manuscripts by Pawlowski et al. [40,53,54,55,56,57] (Figure 3B). This model demonstrates the relationship between AEFs, shape deformations, and membrane destabilization, which can contribute to electroporation and other electric field-induced cell phenomena including electrofusion and electro-destruction. Electroporation, or electropermeabilization, is of particular interest to this review because this process can increase the permeability of the cell membrane, which would permit drugs that cannot normally gain access via the lipid membrane to enter the cells. The application of AEFs causes different types of stress to act on the cell membrane, thereby disrupting its integrity and stability. Based on electrical and geometric parameters of the system, cells either experience shear stress or extensile mechanical stress. In the bioelectrorheological framework, the cell model consists of three parts—the cytoplasm, membrane, and external medium—which are all simplified to be homogeneous wherein the cytoplasm and medium are considered as non-viscous fluids while the membrane is considered an elastic and viscous element [53,57].

Applying AEFs to cells results in shear stress that is related to deformation of the cell’s shape, which allows determination of viscoelastic and rheological membrane parameters; the deformation is dependent on membrane and cytoplasm properties, the external electric field, and external medium [56]. Different types of stress result in different cell membrane changes. For example, isotropic stresses result in volume changes and anisotropic stresses result in shape changes. The bioelectrorheological model specifically defines cell shape deformations as a function of external electric field conditions, electric parameters (of the cytoplasm, cell membrane, and external medium), dielectric lossiness, conductivity of media, and cell membrane viscosity, surface tension, and other rheological parameters of the membrane [40,53,54,55,56,57]. As a result, by analyzing the cell shape deformation induced by AEFs and taking into account cell and membrane viscoelastic and rheological parameters, the membrane shear stress can be calculated. The shear stress is assumed to have developed due to Maxwell stress [53].

Pawlowski and colleagues correlated changes in the cell shape to mean shape deformations of the cell membrane, and in turn correlated these to mean shear stress in the membrane [53]. Consequently, cell deformations were predicted based on electric field frequency; frequencies in the range of 1 to 1,000,000 kHz were tested; these evaluations revealed a threshold frequency at which cell deformations occur, dependent on the aforementioned properties [53]. Shear stress acting on the membrane increased with cell radius, decreased with external medium conductivity, and increased with membrane conductivity [53]. Further testing in *N. crassa* (mold cells) showed that the model was able to predict changes in cell volume and surface caused by AEFs of 3000 kHz [54]. The deformations observed were reversible and non-damaging, similar to the induction of membrane fenestrae by TTFields [27,53]. There are 2 main hypotheses that attempt to explain this reversibility and why it takes time (microseconds) for the membrane to reseal itself. The first claims that electroporation results in conformational changes in the lipids of the membrane that form structures which take time to decay and the second claims that oxidation of the lipids alters membrane properties [58].

The bioelectrorheological model of the cell can also be applied to extensile mechanical stress, which is observed with application of AEFs with frequencies of 0.1 to 10,000 kHz, and leads to destabilization of the cell membrane and, eventually, electroporation. Extensile stress is defined as “the difference between two components of total stress corresponding to extension and compression of the membrane at a given point” [40]. It is influenced by membrane area and thickness as well as position on the membrane [40]. Stress which is calculated using Maxwell-Wagner polarization, Maxwell stress, mechanical normal, and tangential stress reaches maximum values at the poles and high values at the equator of the cell [40]. Furthermore, extensile stress is dependent on the AEF’s amplitude and frequency and the electric and geometric parameters of the cell system, including membrane dielectric properties and cell radius [40]. To analyze extensile stress, the model of the cell is simplified to an elastic shell assumed to be homogeneous with constant volume and slightly compressible area and thickness [40]. Internal and external media are also assumed to be isotropic, noncompressible, non-viscous liquids. Changes in shell thickness, area, and volume (which is assumed to be zero) are used to calculate the magnitude of extensile stress caused by oscillating electric fields [40]. In *N. crassa*, the maximum value of extensile stress was modeled as a function of electric field frequency (ranging from 0.1 to 100,000 kHz) using an electric field strength (E) of 250 V/cm [40], which is two orders of magnitude greater than the field strength of TTFields (i.e., 1–4 V/cm). A graph of these results revealed that the maximum extensile stress remains constantly high at low AEF frequencies, and decreases in a sigmoidal fashion at higher frequencies [40]. However, this range of frequencies that results in high constant extensile stress values can be extended to include even higher frequencies, by increasing the external medium conductivity (Table 1). Thus, external medium conductivity and electric field frequency have a significant influence on the magnitude of stress experienced by the cell, and therefore its membrane’s stability.

This concept of electric field frequency affecting membrane stability has important implications for the mechanism behind electroporation, electrofusion, and electro-destruction. For our purposes of attempting to explain TTFields-induced cell membrane permeabilization, we will focus on electroporation in the next section. However, it is important to note that susceptibility to electro-destruction reaches a maximum value at 100 kHz (which is within the range of TTFields frequencies (100–500 kHz)), and that membrane viscosity decreases with increasing frequency in a sigmoidal fashion, reaching minimum values at 100 kHz [55]. Heat energy increases with increasing frequency and reaches maximal values at frequency ranges where TTFields have optimal anti-cancer efficacy (e.g., 200 kHz for glioblastoma and 150 kHz for pleural malignant mesothelioma [12,13,59]). This is significant because heat energy leads to membrane destabilization, which in turn leads to an increased susceptibility to electroporation [55].

In another study by Pawlowski and colleagues, the simplified model of a cell as a homogenous shell was used to demonstrate the electroporating effects of AEFs on Tib9 mouse myeloma plasma cells [55]. The energy for electroporation in these cells was less than that needed for electro-destruction. Susceptibility to electroporation (s(p)) was represented by the reciprocal of the extensile stress needed for electroporation ($\sigma_{0}^{e}$(p)), i.e., s(p) = ($\sigma_{0}^{e}$(p)) ^−1^ [55]. Although s(p) increased with increasing AEF frequency, it was found that the extent of increased susceptibility became attenuated at higher conductivities of the external medium (Table 2) [55]. However, this range of frequencies that result in high extensile stress values can be extended to include even higher frequencies by increasing the conductivity of the external medium [55]. These findings align with the previous findings that high magnitudes of stress stayed constant for wider ranges of AEF frequencies when external medium conductivity was increased; this may have important implications on the effects of pH levels on the electroporation of cancer cells. Table 3 summarizes the parameters and terms involved in the bioelectrorheological model of Pawlowski et al. [40,53,54,55,56,57].

**Figure 4:**
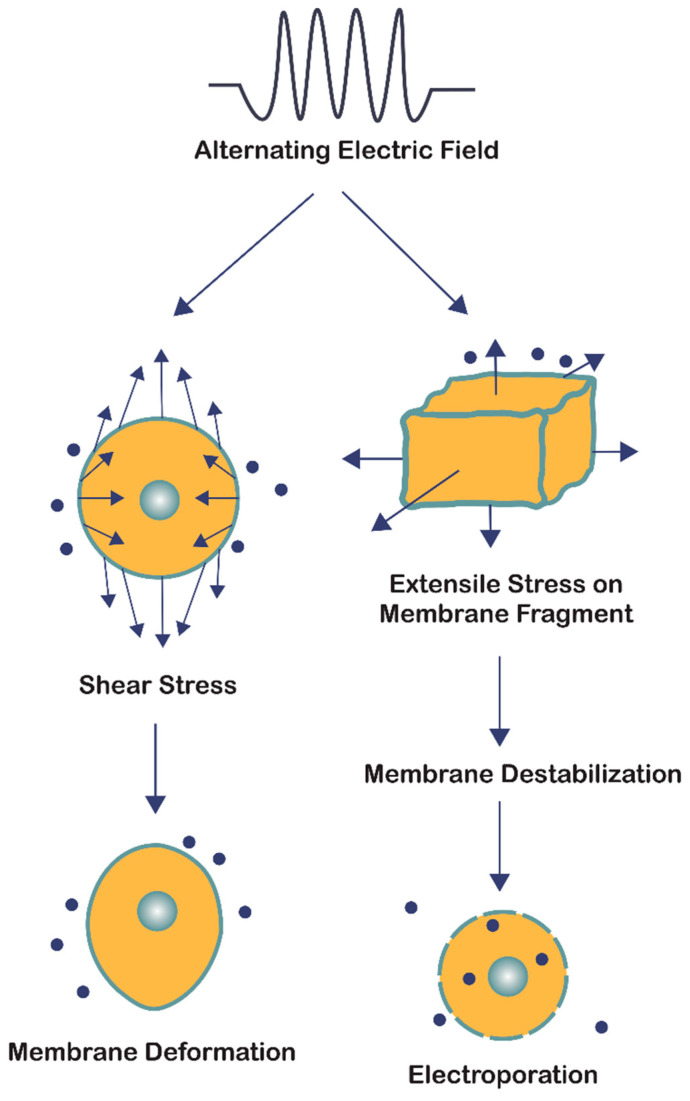
The bioelectrorheological model in which exogenously applied electric fields may shape deformations and destabilize membranes, which can contribute to electroporation and other electric field-induced cell phenomena including electrofusion and electro-destruction.

In summary, the bioelectrorheological model addresses two forms of cell stress resulting from the application of AEFs: shear stress and extensile stress (Figure 4). Both are influenced by the electric and geometrical parameters of the system; however, they affect the cell membrane in two distinct ways. Shear stress leads to physical deformations of cell shape and form, while extensile stress reversibly destabilizes the membrane thereby leaving the cell vulnerable to electroporation, electrofusion, and electro-destruction [56]. Although the electric field strengths studied by Pawlowski and colleagues [40,53,54,55,56,57] were two orders of magnitude greater than that of TTFields, the bioelectrorheological model provides a possible explanation to the causes of electroporation by demonstrating the combined effects of heat energy and extensile stress leading to reversible cell membrane destabilization, which can directly result in electroporation. Further testing of this model on cancer cells and modifying the model to take into account contact with nearby cells and cell inhomogeneities will provide deeper insights.

### 2.3. Electroporation Model

**Figure 5:**
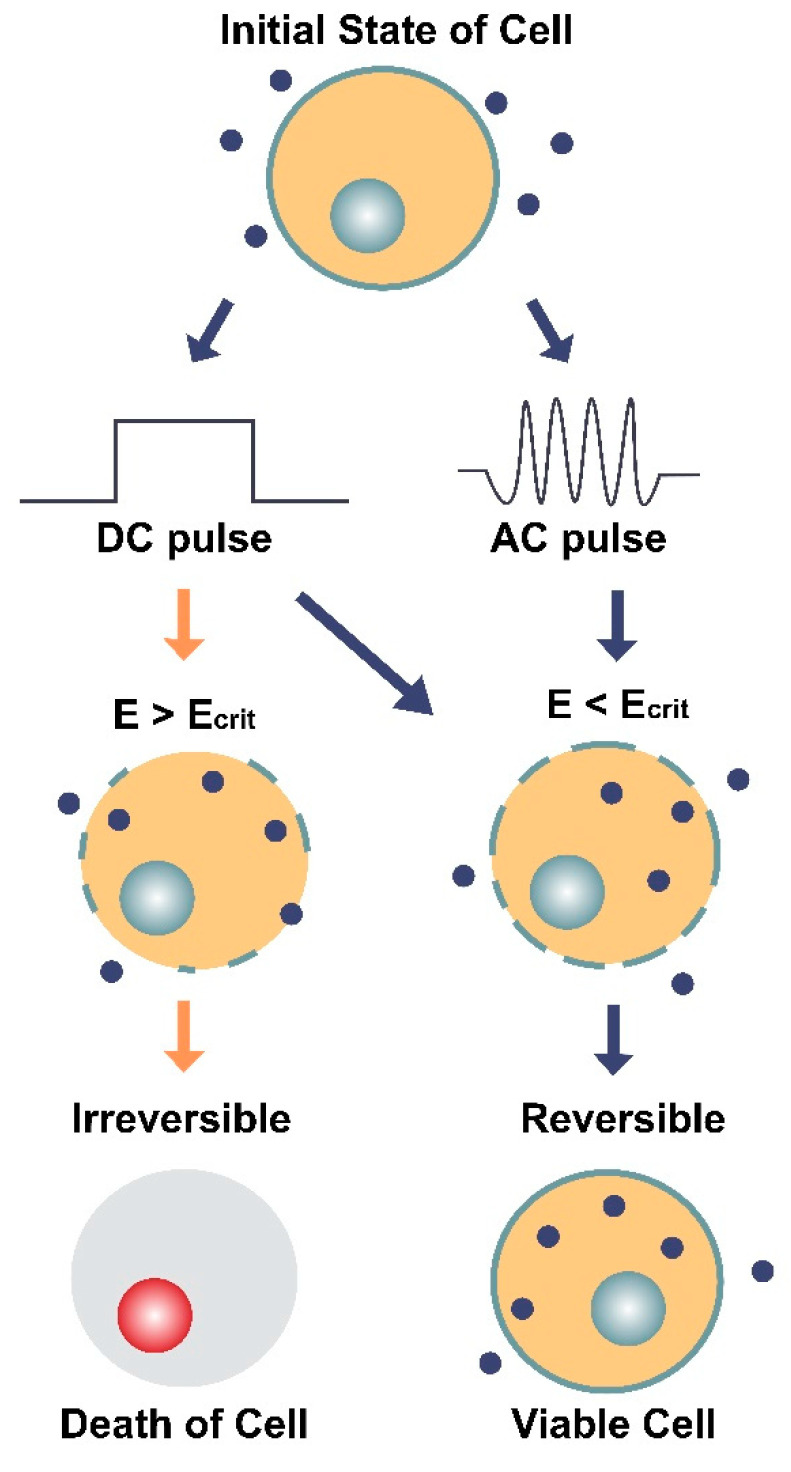
Illustration of the two main types of electroporation (direct current [DC] and alternating current [AC]) and their effects on a cell and its membrane. DC involves the use of short, individual pulses of electric charge whereas AC applies electric charge in an oscillating motion (increasing and decreasing) over a period of time. The dark blue circles represent molecules (average Stokes diameter ~20 nm [64]) that can only enter the cell through pores in the membrane. E is the electric field intensity (units V/cm) and E_crit_ is the minimum field intensity required to reach the cell’s membrane potential threshold. The diagram shows how E can impact the cell’s survival depending on the type of current (DC or AC) and whether E is greater or less than the critical field intensity (E_crit_). When E < E_crit_, the effects of electroporation are reversible, and the cell remains viable.

AEFs have been shown to permeabilize cancer cell membranes in a selective and reversible manner [27]. Electroporation is the use of high-intensity (250–3000 V/cm) DC or AC electric field pulses to cause membrane destabilization, thereby resulting in pore formation (Figure 1, Figure 3C and Figure 5) [60,61,62]. Electroporation pulses can range from nanoseconds to milliseconds in duration and tend to form aqueous pores in the plasma membrane that are on the order of ~1 nm in radius [60,62,63,64]. Whereas DC electroporation is the use of a singular pulse within a specified time interval, AC electroporation involves delivering pulses through an oscillating electric field that is characterized by its frequency (ƒ) [61].

Both types of electroporation are believed to cause a dielectric breakdown of the cell. However, the oscillating nature of AC electroporation is thought to provoke structural fatigue of the membrane [61], comparable to the extensile stress proposed in the bioelectrorheological model [40,53,54,55,56,57]. When both kinds of pulses (DC and AC) are applied together at their optimal settings, which involves 40 kHz frequency for the AC component, both transfection efficiency and cell survivability have been shown to increase [61].

Electroporation’s ability to cause transfection is one of the most studied and widely used applications of electric field pulses. While it has been recognized for some time now that aqueous pore formation allows for increased permeability across the plasma membrane, recent studies suggest that chemical and structural changes in the lipids and proteins (such as ion channels) of the membrane itself also contribute to the mechanism of action [62]. An important component of this proposed mechanism, by which altering the plasma membrane through electric pulses leads to increased permeability, is that there are two “thresholds” which are not universal but rather particular to the parameters being used (i.e., cell type/size, membrane curvature, exposure time, temperature, transported molecule, and osmotic pressure) [62]. The first threshold is the induced transmembrane potential (V_m_) needed to cause the detectable increase in membrane permeability and the second threshold (critical V_m_) is the point at which reversible electroporation instead becomes irreversible [62].

After the first threshold is surpassed, it is thought that the V_m_ depolarizes the membrane sufficiently to trigger the formation of aqueous pores and stimulate other voltage-dependent processes such as ion channel activation and cytoskeletal disruption [62,65]. Researchers have often used calcium concentration as a measure of permeability since this ion has a greater extracellular concentration at physiological conditions. In a study by Craviso and colleagues, the activation of Ca_v_^2+^ via DC electroporation was confirmed using Ca_v_^2+^ inhibitors to identify the specific L-type channel being used and confirm the calcium influx was due to channel permeabilization and not aqueous pore formation [65]. This study concluded that 5-ns duration, high-intensity (50,000 V/cm) electric pulses were sufficient to open up the L-type Ca_v_^2+^ channels, resulting in detectable calcium influx [65].

To better understand the effects that electroporation can have on the cell cytoskeleton, Kanthou and colleagues studied endothelial cells with fluorescence imaging and found that at electric field strengths in the 50–200 V/cm range, electroporation of endothelial cells is not only possible, but imaging also revealed significant, reversible disruption of interphase microtubules and actin filaments [66]. Based on these results, the scientists hypothesized that the damaging effect of electroporation could last longer in tumor cells, given differences in membrane and cytoskeletal composition, and helped elucidate why this therapy selectively affects tumor cells [66].

Another effect through which electroporation has been found to target cancer cells is tumor ablation. In tumor ablation, cells are exposed to an electric field intensity that results in a V_m_ that surpasses the abovementioned second threshold (critical V_m_). The critical V_m_ is cell-specific and is the transmembrane potential that destabilizes the membrane beyond repair (i.e., irreversible electroporation), resulting in cell death [41]. Various studies have shown the effectiveness of using irreversible electroporation to induce tumor ablation and suggest that its ability to target malignant cells is due to plasma membrane differences between cancer and non-cancer cells [67,68]. The V_m_ of both non-cancer and cancer cells depolarizes during proliferation, to about −15 mV, but post-mitotic non-cancer cells return to a typical resting V_m_ of −70 mV whereas post-mitotic cancer cells achieve a resting V_m_ of −25 mV [51,69]. The depolarized resting V_m_ in cancer cells relative to that in non-cancer cells is thought to be caused by altered lipid and sterol membrane composition, which results in an influx of sodium ions into the cell and a collection of negative charges on the cell coat [70]. Modulation of chloride, sodium, potassium, and calcium channel activity has also been found to contribute to the relatively depolarized resting V_m_ in cancer cells [71]. For example, digitalis, a sodium-potassium ATPase inhibitor, has been shown to limit the proliferation of human breast cancer cells [72]. The consequence of this difference is that the resting V_m_ needed to reach both the first and second thresholds is lower in cancer cells than in non-cancer cells. The relatively depolarized resting V_m_ in cancer cells [28,29,30,31,73,74,75] could explain why, compared to non-cancer cells, cancer cells are more vulnerable to both electroporation and TTFields [67,69] (see Table S4). Table 4 compares the parameters between TTFields and electroporation (DC and AC).

Similarly, electrochemotherapy aims to damage cancer cells, but it does so by porating the membrane so that chemotherapeutic agents can more easily permeate into the cell, resulting in a synergistic effect [79]. In particular, electrochemotherapy has proven effective in targeting cancer cells that are otherwise resistant to the chemotherapy being administered [78]. For instance, calcium electroporation is thought to work by the same mechanism as electrochemotherapy, and it uses calcium because of its role as a second messenger for various processes resulting in cell death [78]. A study by Frandsen and colleagues investigated the mechanisms of calcium electroporation, leading the authors to hypothesize that cancer cells may be more sensitive to electroporation because they are less equipped to repair and restore the plasma membrane after damage [78,80].

The results from electroporation studies further support that ion channel activation and ion influx, and perhaps other effects such as cytoskeleton damage, can be used as biomarkers of responsiveness to TTFields, given that these phenomena are observed after exposure to electric fields [27,46,62,65]. While the electroporation model can provide further insight into how electric fields affect cancer cells and their membranes, we believe significant differences in parameters (i.e., electric field intensity, electric field frequency, and duration of exposure) limit the degree to which electroporation is comparable to TTFields. The electroporation model can be used to predict the effects of TTFields on cancer and non-cancer cells, but it does not fully explain the mechanism by which TTFields leads to cell membrane permeabilization. A comparative study on transmembrane pores induced by TTFields versus electroporation will help to clarify the extent of overlap between the mechanisms of action of these two modalities. Further evidence of the relatively depolarized resting V_m_ of cancer cells in comparison to that of non-cancer cells has been observed in various cellular types of the brain (summarized in Table S4) [81,82,83].

## 3. Discussion

Recent investigations [27] have shown that the standard theory of how TTFields work [8] requires further elaboration. The standard theory proposes a disruption of the mitotic spindle by TTFields in actively dividing cells, thereby leading to aberrant cellular division and eventual cellular death [8,84]. Mitotic disruption by exogenously imposed electric fields have been reported [8]; however, mitotic spindles are not the only biological structures that possess intrinsic dipole moments. To illustrate, DNA itself possesses intrinsic dipole moments and recent investigations have revealed that TTFields may have effects on DNA damage response and replication stress, ER stress, membrane permeability, autophagy, and immune response [5]. In addition, recent modeling studies in the context of dipole alignment suggest that the magnitude of the electric field caused by TTFields may not be sufficient to overcome Brownian motion inherent within the cytoplasm of single cancer cells [51,69]. TTFields have been shown to affect other cellular structures. For example, the application of alternating electric fields has been shown to disrupt the membranes of subcellular compartments [85] as well as the plasma membrane of human glioblastoma cells [27]. The latter may have important clinical implications since enhanced membrane permeability of cancer cells via TTFields may render those cells more sensitive to chemotherapeutic treatments (e.g., temozolomide) when combined with TTFields [12,86,87,88,89]. Such a phenomenon may explain our recent findings of synergistic activity between TTFields and the potential chemotherapeutic agent, Withaferin A [26]. The ability to enhance the efficacy of chemotherapeutics may be explained by the induction of fenestrae in the plasma membrane by TTFields, which we previously observed with scanning electron microscopy [27].

We thus embarked upon theoretical investigations to explain the observed effects of TTFields and reviewed three possible explanatory models: (1) voltage-gated ion channels, (2) the bioelectrorheological model, and (3) electroporation. Each model is related to and possesses features that are consistent with the reported effects of TTFields. For example, TTFields have been reported to activate Ca_v_^2+^ channels [46], membrane distortions predicted by the bioelectrorheological model are likely another feature of TTFields’ actions [40], and membrane fenestration is a hallmark of reversible electroporation [62]. However, as reviewed above, the functionally therapeutic ranges of TTFields’ frequency (100–500 kHz) and field strength (1–4 V/cm) often do not overlap with the proposed field strengths and frequencies of the Ca_v_^2+^, bioelectrorheological, and electroporation models. Further dedicated studies would help to resolve these gaps and provide a more complete understanding of the membrane permeabilizing effects of TTFields.

The resolution of the observed versus theoretical discrepancies may stem from the fact that most computational modeling studies are based upon single cells in isolation. Modeling of the arrangement of cell clusters and how such configurations lead to inhomogeneous electric fields and/or the amplification of their effects on the cellular membrane should be pursued. Augmentation of field strength in adjacent membrane regions of densely packed cells (i.e., the tissue level) may lead to values that would be concordant with either the bioelectrorheological or electroporation models. Indeed, research by some of us (co-authors TM and ZB, personal communication) shows that finite element modeling predicts that when cells are in close proximity to each other, electric field lines are concentrated between the contact points in a frequency-dependent manner [51,69]. The unexpectedly high field strengths achieved in these regions may remove the obstacle of electric field strength being insufficient for bioelectrorheological and electroporative processes to be able to explain the empirical observation of membrane fenestration due to TTFields [27]. Since resting cancer cells are relatively depolarized compared to non-cancer cells, the same phenomenon likewise provides an explanation for TTFields’ effect on cancer but not non-cancer cells as the former needs less field strength concentrations to trigger an effect. Certainly, the recent work of Li et al. [51,69] suggests that the membrane potential could serve as an important read-out to monitor in studies of TTFields. This may not be surprising given that the resting V_m_ of cancer cells is depolarized relative to that of non-cancer cells [28,29,30,31,73,74,75].

Future studies should also consider varying the ionic strength of the extracellular media to determine if such alterations could impact electric field distribution on the cellular membrane. Such an approach may reveal how the tumor microenvironment influences the distribution of AEFs on the membrane. Membrane-bound fluorescent or luminescent probes as well as electron microscopy investigations of cellular and subcellular membranes [27] could be used to validate the modeling results from such investigations. Calcium influx and Ca_v_^2+^ activation might also serve as novel biomarkers of the efficacy of TTFields [46].

The increased cellular proliferation and density in cancer tissues could explain both the high field strength needed for the AEF effects described herein as well as why AEFs affect cancer but not non-cancer cells. Our modeling also indicates that using microfluidics to measure shear stress (τ) induced by TTFields and investigations into TTFields-induced alterations in transmembrane fenestration number, distribution, and size are pertinent to cancer research. Although exogenously imposed AEFs are not specifically tied to a singular signal transduction pathway in cancer cells, we have recently reported that phosphatase and tensin homolog (PTEN) mutations predict benefit from TTFields in patients with recurrent isocitrate dehydrogenase (IDH) wild-type GBM [90], which represents the first molecular biology-based predictor of responsiveness to TTFields therapy. PTEN is involved in maintaining mitotic spindle architecture and promoting chromosome alignment and segregation, functions overlapping with the postulated mechanism of action of TTFields [8,91,92]. PTEN mutation leads to disruption of proper spindle assembly and chromosome segregation, which results in mitotic catastrophe [93,94]. Thus, it is possible that loss-of-function mutations in PTEN could potentiate the inhibition of microtubule polymerization and mitotic spindle apparatus assembly that are known to result from TTFields exposure. Ultimately, future investigations should provide insights towards a unified understanding of the mechanism of action of TTFields-induced cytoskeletal changes, cancer cell membrane permeabilization, and permeabilization of the blood-brain barrier [95].

## 4. Conclusions

TTFields is a form of alternating electric fields (AEF) therapy and is emerging as the fourth approved therapy (after surgery, radiation, and chemotherapy) in patients with glioblastoma. Despite its demonstrated clinical efficacy, TTFields’ mechanisms of action is not yet fully elucidated. This review summarized the current literature to provide a broader understanding of AEF’s actions on cell membranes. It provided an overview of three mechanistic models (the voltage-gated ion channel, bioelectrorheological, and electroporation models) that may explain the recent observations of AEFs’ effects on membrane permeability. The effects of AEF on membrane function in cancers is insufficiently explained by the impact on voltage-gated calcium channels alone; however, alterations in channel function might serve as a biomarker of AEF action. Rather, the explanation of AEF-induced alteration of membrane function will most likely consist of a combination of the bioelectrorheological and electroporation models. In our investigations, inconsistencies were noted in both the effective frequency range and field strength between TTFields versus all three proposed models. Through theoretical investigations, we addressed the inhomogeneities of electric fields on cellular membranes as a function of disease state, external microenvironment, and tissue or cellular organization. These findings could be exploited to enhance drug delivery to cancer cells shielded by the blood–brain barrier. Future experimental strategies for validation were outlined.

## Figures and Tables

**Table 1:** Maximal extensile stress values ($\sigma_{0}^{e}$, units N/m^2^), measured at different electric field frequencies (ƒ, units kHz) in *N. crassa* mold cells with various external medium conductivities (Re(k), units mS/m). Based on data from Pawlowski et al. [40].

Re(k):	1 mS/m	2 mS/m	5 mS/m	10 mS/m	20 mS/m	50 mS/m	200 mS/m
ƒ (kHz)	$\sigma_{0}^{e}$(N/m^2^)						
0.1	9.1 × 10^4^	9.1 × 10^4^	9.1 × 10^4^	9.1 × 10^4^	9.1 × 10^4^	9.1 × 10^4^	9.1 × 10^4^
1	7.8 × 10^4^	8.8 × 10^4^	9.0 × 10^4^	9.1 × 10^4^	9.1 × 10^4^	9.1 × 10^4^	9.1 × 10^4^
10	6.9 × 10^3^	2.2 × 10^4^	6.0 × 10^4^	8.3 × 10^4^	8.9 × 10^4^	9.1 × 10^4^	9.2 × 10^4^
100	5.8 × 10^2^	5.8 × 10^2^	2.2 × 10^3^	6.4 × 10^3^	1.7 × 10^4^	4.8 × 10^4^	7.6 × 10^4^
1000	5.8 × 10^2^	5.8 × 10^2^	5.8 × 10^2^	5.8 × 10^2^	5.8 × 10^2^	1.3 × 10^3^	3.6 × 10^3^

**Table 2:** Susceptibility to electroporation (s(p), units m^2^/N), defined as ($\sigma_{0}^{e}$ (p)) ^−1^ where $\sigma_{0}^{e}$ (p) (units N/m^2^) is the extensile stress needed for electroporation, measured at different frequencies (ƒ, units kHz) in Tib9 mouse myeloma plasma cells in external medium with varying conductivities (Re(k), units mS/m). Based on data from Pawlowski et al. [55].

Re(k):	2 mS/m	14 mS/m	42 mS/m
ƒ (kHz)	S(p) (m^2^/N)		
0.1	2.0 × 10^−5^	1.1 × 10^−5^	1.0 × 10^−5^
1	1.9 × 10^−5^	1.1 × 10^−5^	1.0 × 10^−5^
10	1.7 × 10^−5^	9.9 × 10^−6^	1.0 × 10^−5^
100	~3.7 × 10^−5^	1.4 × 10^−5^	7.7 × 10^−6^

**Table 3:** List of parameters and terms and how they relate to the bioelectrorheological model of Pawlowski et al. [40,53,54,55,56,57]. ↑ indicates increased, ↓ indicates decreased.

Parameter or Term	Relevance to Bioelectrorheological Model
Conductivity of external medium (Re(k), units mS/m)	↑ Re[k] causes ↑ extensile stress and consequent ↓ susceptibility to electroporation
Extensile stress ($\sigma_{0}^{e}$, units N/m^2^)	Causes destabilization of cell membrane, which can eventually cause electroporation
Extensile stress needed for electroporation ($\sigma_{0}^{e}$(p), units N/m^2^)	Causes electroporation, ↑ ƒ leads to ↓ $\sigma_{0}^{e}$(p)
Frequency (ƒ, units Hz or kHz) of alternating electric field (AEF)	↑ ƒ causes ↓ $\sigma_{0}^{e}$(p)
Radius of cell (r, units µm)	↑ cell radius causes ↑ shear stress
Reversibility	Formation of pores and membrane damage are transient
Shear stress (τ, units Pa)	Leads to physical deformations of cell shape. ↑ cell radius or ↑ membrane conductivity cause ↑ τ; ↑ Re(k) causes ↓ τ
Susceptibility to electroporation (s(p), units m^2^/N), defined as ($\sigma_{0}^{e}$(p)) −1	s(p) varies nonlinearly with ƒ, see reference [55]; ↑ Re(k) causes ↓ s(p)

**Table 4:** Comparison of parameters between tumor treating fields (TTFields) and electroporation (direct current (DC) and alternating current (AC)).

Parameter	TTFields	Electroporation	
		DC	AC
Duration of electric field exposure (t)	Days (in vitro) [27] or months (in patients) [12]	Micro- to milli- seconds (optimal at 10 μs) [60]	Micro- to milli- seconds (optimal at 2 ms) [61]
Frequency (ƒ, units kHz) of electric field	200 [27]	N/A	40 [60]
Intensity (or strength) of electric field (E, units V/cm)	1–4 [27,76]	250–300 [60]	500–5000 [61]
Pore Size (units nm)	17.5 ± 10.8 (average diameter) [27]	25–120 (average diameter) [61]	Unknown
Reversibility of membrane permeabilization (t_R_)	Minutes to days [27,46]	Seconds to minutes [62]	Unknown
Schwan Equation	$\Delta V=f_{s}E_{e}R\cos\theta \frac{1}{1+j\omega \tau_{m}}$ [69]	$V_{m}=1.5rEcos\theta$ [61]	$V_{m}=\frac{1.5rEcos\theta }{{\left[1+{\left(\omega \tau \right)}^{2}\right]}^{1/2}}$ [77]
Effects			
Membrane permeabilization?	Yes (reversible) [27]	Yes (reversible) [61,62]	Yes (reversible) [61]
Ion channel activation?	Yes (reversible) [46]	Yes (reversible) [65]	Unknown
Cytoskeletal damage?	Yes [8]	Yes (reversible) [66]	Unknown
Tumor Ablation?	No [36]	Yes [41]	Unknown
Synergistic electrochemo-therapy?	Yes [26]	Yes [78]	Unknown

## Supplementary Materials

The following are available online at https://www.mdpi.com/article/10.3390/cancers13092283/s1, Table S1. Parameters of 4-stage model of voltage-gated ion channels, Table S2. Parameters of bioelectrorheological model, Table S3. Parameters of electroporation model, Table S4. Differences in resting membrane potential (V_m_) between cancer and non-cancer cells.
