# Supplementary material for: Permeabilizing Cell Membranes with Electric Fields
Source: Cancers (Basel). 2021 May 10;13(9):2283. doi: 10.3390/cancers13092283 (PMC8126200; doi:10.3390/cancers13092283)
Supplement: Supplementary file 1 [file cancers-13-02283-s001.zip › cancers-1169693-supplemental-tables/20210314 Table S1_final.pdf]

SUPPLEMENTARY TABLE S1. Parameters of 4-stage model of voltage-gated ion channels

Estimated model parameters using the patch-clamp technique

Rate Constant	Wild-type control	Wild-type FPL	IIS4N control	IIS4N FPL	A780T control	A780T FPL
α	0.27 ± 0.06	0.26 ± 0.01	0.16 ± 0.03	0.08 ± 0.02	0.24 ± 0.09	0.09 ± 0.04
β	0.21 ± 0.04	0.07 ± 0.03	0.31 ± 0.07	0.14 ± 0.02	0.61 ± 0.08	0.22 ± 0.05
γ	0.02 ± 0.01	0.002 ± 0.001	0.03 ± 0.01	0.003 ± 0.001	0.01 ± 0.01	0.014 ± 0.006
δ	2.09 ± 1.05	2.68 ± 1.00	2.48 ± 1.23	1.36 ± 0.81	1.85 ± 0.29	1.45 ± 0.12
x0	1.65 ± 0.80	7.77 ± 3.41	1.35 ± 0.50	1.33 ± 0.38	2.81 ± 0.97	57.14 ± 14.63
y0	0.21 ± 0.07	0.17 ± 0.09	0.16 ± 0.04	0.02 ± 0.003	0.0008 ± 0.0003	0.00018 ± 0.00003
kx	12.39 ± 3.12	5.67 ± 0.82	10.42 ± 2.29	8.07 ± 0.74	16.84 ± 1.82	5.85 ± 0.20
ky	51.61 ± 37.06	25.32 ± 5.31	74.70 ± 33.78	29.72 ± 4.32	7.08 ± 0.79	12.30 ± 0.90
G	19.68 ± 6.72	60.86 ± 9.90	22.47 ± 9.68	40.03 ± 9.32	108.24 ± 36.55	330.04 ± 92.47

FPL: In the presence of Calcium agonist FPL 64174 (1 μM)
Reference: https://pubmed.ncbi.nlm.nih.gov/25966699/

Rate constants of A↔O and R↔D transitions (in ms^−1), estimated using data from Hohaus et al. (2005). (shown below)

Construct	α	β	γ	δ
Wild-type[a]	0.15 ± 0.11	0.24 ± 0.08	0.021 ± 0.021	1.39 ± 0.17
G770P	0.33 ± 0.31	0.31 ± 0.21	0.030 ± 0.073	0.56 ± 0.11
N771P[a]	0.95 ± 0.63	0.11 ± 0.07	0.111 ± 0.057	1.41 ± 0.23
F778P[a]	0.13 ± 0.12	0.28 ± 0.09	0.016 ± 0.026	0.70 ± 0.09
L779P[a]	1.79 ± 0.81	0.11 ± 0.02	0.002 ± 0.002	0.36 ± 0.09
A780P[a]	0.19 ± 0.03	0.0064 ± 0.0022	0.015 ± 0.005	0.14 ± 0.02
A780G	0.96 ± 0.62	0.27 ± 0.12	0.037 ± 0.022	2.10 ± 0.33
I781T[a]	1.95 ± 0.91	0.045 ± 0.012	0.009 ± 0.003	1.02 ± 0.26
I781P[a]	4.16 ± 1.86	0.034 ± 0.007	0.003 ± 0.001	0.74 ± 0.43
I781G	1.73 ± 0.81	0.036 ± 0.010	0.011 ± 0.003	0.78 ± 0.18
I781A[a]	1.72 ± 0.79	0.132 ± 0.028	0.003 ± 0.001	1.55 ± 0.44
I781V[a]	0.55 ± 0.30	0.22 ± 0.03	0.0003 ± 0.0002	2.01 ± 0.77
I781N[a]	2.17 ± 0.99	0.10 ± 0.02	0.003 ± 0.002	0.74 ± 0.20
A782P[a]	2.65 ± 1.23	0.111 ± 0.026	0.004 ± 0.002	0.94 ± 0.27
A782G	1.74 ± 0.97	0.32 ± 0.10	0.013 ± 0.009	1.96 ± 0.37
D784G	0.88 ± 0.53	0.34 ± 0.09	0.008 ± 0.006	1.62 ± 0.35

[a]: Rate constants determined using data from Hohaus et al. (2005). https://pubmed.ncbi.nlm.nih.gov/16157588/
Reference: https://www.ncbi.nlm.nih.gov/pmc/articles/PMC2737230/

The influence of IIS6 pore mutations on voltage-dependent gating of CaV1.2

Midpoints and slope factors of the activation curves and amount of channel inactivation during a 300-ms pulse (r300) to the peak potentials of the I–V curve.

	Half-activation	Slope	*r300*
	mV	*mV*	%
**Co-expression of β^{1}[a]**			
Wild-type	–9.9 ± 1.1	6.3 ± 0.7	65 ± 4
I781T	–37.7 ± 1.2	7.2 ± 1.0	47 ± 3

I781P	–47.2 ± 1.1	6.3 ± 0.6	53 ± 6
I781L	–20.6 ± 1.2	6.1 ± 0.2	63 ± 7
I781A	–29.5 ± 0.6	7.9 ± 0.5	51 ± 4
I781N	–34.6 ± 1.6	9.1 ± 0.4	37 ± 6
I781Q	No currents	N/A	N/A
I781R	No currents	N/A	N/A
C769P	2.8 ± 0.8	4.9 ± 1.0	67 ± 7
G770P	–11.4 ± 1.3	5.8 ± 0.3	62 ± 4
N771P	–26.9 ± 1.1	4.9 ± 0.2	43 ± 4
Y772P	No currents	N/A	N/A
I773P	–14.2 ± 1.8	2.9 ± 0.6	65 ± 6
L774P	No currents	N/A	N/A
L775P	No currents	N/A	N/A
N776P	No currents	N/A	N/A
V777P	No currents	N/A	N/A
F778P	–9.3 ± 0.8	7.7 ± 0.3	23 ± 3
L779P	–29.78 ± 1.90	6.0 ± 0.4	3 ± 1
A780P	–37.1 ± 0.7	5.7 ± 0.8	2 ± 1
A782P	–35.7 ± 1.4	9.8 ± 0.3	30 ± 5
V783P	No currents	N/A	N/A
**Co-expression of β^{2a}**			
Wild-type	–13.8 ± 0.9	6.0 ± 0.5	16 ± 5
I781T	–44.9 ± 1.4	8.8 ± 1.0	25 ± 4
I781P	–52.3 ± 1.3	7.3 ± 0.8	27 ± 6

β^{1a}: β subunit of a DHP-sensitive calcium channel in skeletal muscle with the DNA sequence from Perez-Reyes et al. 1989 (https://pubmed.ncbi.nlm.nih.gov/2549640/)
β^{2a}: β subunit of a DHP-receptor/calcium channel in skeletal muscle with the DNA sequence from Perez-Reyes et al. 1992 (https://pubmed.ncbi.nlm.nih.gov/1370480/)
Reference: https://pubmed.ncbi.nlm.nih.gov/16157588/

Model parameters describing the R↔A transition

Rate constant	Estimated value
x0 (ms^−1)	0.427 ± 0.029
kx (mV)	328.8 ± 447.4
y0 (ms^−1)	0.216 ± 0.084
ky (mV)	9.04 ± 0.52

Reference: https://www.ncbi.nlm.nih.gov/pmc/articles/PMC2737230/

Rate constants of pore opening and closure

	α	β	γ	δ
Wild-type	0.2	0.25	0.08	1.2
1781T	8.3	0.07	0.011	0.6
1781P	22	0.035	0.007	0.3

Parameters for voltage dependent rate con- stants x(V) and y(V)

Rate constant	Estimated value
x0 (ms^−1)	0.38

kx (mV)	55
y0 (ms^−1)	0.17
ky (mV)	8

Reference: https://www.ncbi.nlm.nih.gov/pmc/articles/PMC3196840/

Midpoints and slope factors of the activation curve of CaV1.2 IIS6 mutants

Mutant	Half-activation	Slope
	mV	***mV***
Wild-type[a]	−9.9 ± 1.1	6.3 ± 0.7
A780P[a]	−37.1 ± 0.7	5.7 ± 0.8
A780G	−19.3 ± 0.8	6.3 ± 0.6
I781P[a]	−47.2 ± 1.1	6.3 ± 0.6
I781G	−40.2 ± 0.8	7.5 ± 0.7
I781T[a]	−37.7 ± 1.2	7.2 ± 1.0
A782P[a]	−35.7 ± 1.4	9.8 ± 0.3
A782G	−22.2 ± 0.4	7.8 ± 0.4
V783G	No current	N/A
D784G	−15.6 ± 1.1	6.3 ± 0.6

a: Data from Hohaus et al. (2005). https://pubmed.ncbi.nlm.nih.gov/16157588/

Reference: https://www.ncbi.nlm.nih.gov/pmc/articles/PMC2737230/

S4 Segments of Cav 1.2 Channels: Midpoints and slope factors (k) of the activation curves and estimated effective charge (αZ)

Mutant	V0.5, mV	*k act, mV*	αZ
Wild-type	−18.6 ± 0.8 (n = 7)	5.9 ± 0.9	4.27 ± 0.65
G432W[b]	−33.7 ± 0.6 (n = 8)	4.7 ± 0.4	5.36 ± 0.46
A780T[b]	−44.0 ± 0.6 (n = 5)	5.1 ± 0.6	4.94 ± 0.58
G1193T[b]	−49.8 ± 1.0 (n = 7)	5.1 ± 0.6	4.94 ± 0.58
A1503G[b]	−41.4 ± 0.8 (n = 5)	5.8 ± 0.5	4.34 ± 0.37
**IS4 neutralisations**			
IS4N	No detectable current		
K264Q/R267Q/R270Q/R273Q (IS4N+R276)	−33.1 ± 1.1 (n = 5)	8.5 ± 0.6	2.96 ± 0.21
K264Q/R267Q/R270Q (IS4N+R276+R273)	−16.3 ± 0.7 (n = 4)	9.9 ± 0.7	2.54 ± 0.18
G432W/R267Q	−11.9 ± 1.0 (n = 4)	8.5 ± 0.8	2.96 ± 0.28
G432W/IS4N+R276	No detectable current		
A780T/R267Q	−28.5 ± 1.3 (n = 4)	9.6 ± 0.7	2.62 ± 0.19
A780T/IS4N+R276	−42.7 ± 1.1 (n = 4)	11.9 ± 1.0	2.12 ± 0.18
A780T/K264Q/R267Q/R270Q (A780T/IS4N+R276+R273)	−33.5 ± 0.6 (n = 4)	11.5 ± 0.6	2.19 ± 0.11
G1193T/R267Q	−28.6 ± 0.9 (n = 5)	9.1 ± 0.5	2.77 ± 0.15
G1193T/IS4N+R276	No detectable current		
A1503G/R267Q	−28.5 ± 0.6 (n = 5)	9.1 ± 0.4	2.77 ± 0.12
A1503G/IS4N+R276	−41.1 ± 0.6 (n = 5)	8.7 ± 0.5	2.89 ± 0.17
**IIS4 neutralisations**			
IIS4N[a]	−17.5 ± 0.8 (n = 11)	6.7 ± 0.7	3.76 ± 0.39
R650Q/R653Q/R656Q/K659Q (IIS4N+R662)	−23.9 ± 1.0 (n = 6)	6.3 ± 0.7	4.00 ± 0.44

R650Q/R653Q/R656Q (IIS4N+R662+K659)	−18.2 ± 0.7 (n = 5)	7.2 ± 0.6	3.50 ± 0.29
R650Q/R653Q	−25.1 ± 0.9 (n = 5)	5.7 ± 0.6	4.42 ± 0.47
G432W/R656Q	−17.9 ± 0.9 (n = 5)	6.0 ± 0.7	4.20 ± 0.49
G432W/IIS4N+R662	−31.0 ± 0.6 (n = 5)	8.4 ± 0.8	3.00 ± 0.29
G432W/IIS4N[a]	−19.3 ± 0.7 (n = 8)	5.8 ± 0.7	4.34 ± 0.52
A780T/R650Q	−30.7 ± 0.3 (n = 4)	5.7 ± 0.3	4.42 ± 0.23
A780T/R653Q	−30.2 ± 0.6 (n = 5)	5.3 ± 0.3	4.75 ± 0.27
A780T/R656Q	−33.5 ± 0.5 (n = 5)	6.5 ± 0.3	3.88 ± 0.18
A780T/K659Q	−32.0 ± 1.3 (n = 4)	7.1 ± 0.8	3.55 ± 0.40
A780T/R662Q	−34.0 ± 0.7 (n = 5)	5.4 ± 0.3	4.67 ± 0.26
A780T/IIS4N+R662	−33.6 ± 0.6 (n = 4)	6.3 ± 0.3	4.00 ± 0.19
A780T/IIS4N+R650	−39.4 ± 0.8 (n = 4)	6.1 ± 0.6	4.13 ± 0.41
A780T/IIS4N[a]	−32.0 ± 0.7 (n = 10)	5.1 ± 0.7	4.94 ± 0.68
G1193T/R650Q	−39.0 ± 0.9 (n = 5)	6.8 ± 0.6	3.71 ± 0.33
G1193T/R653Q	−34.0 ± 0.6 (n = 6)	6.6 ± 0.5	3.82 ± 0.29
G1193T/R656Q	−38.3 ± 0.8 (n = 5)	5.0 ± 0.5	5.04 ± 0.50
G1193T/K659Q	−40.3 ± 0.9 (n = 4)	4.9 ± 0.5	5.14 ± 0.52
G1193T/R662Q	−40.2 ± 0.4 (n = 6)	5.7 ± 0.3	4.42 ± 0.23
G1193T/IIS4N+R662	−38.0 ± 0.8 (n = 5)	6.1 ± 0.6	4.13 ± 0.41
G1193T/IIS4N[a]	−20.8 ± 0.7 (n = 5)	7.5 ± 0.6	3.36 ± 0.27
A1503G/R656Q	−26.6 ± 0.7 (n = 5)	7.3 ± 0.5	3.45 ± 0.24
A1503G/IIS4N+R662	−25.3 ± 1.0 (n = 6)	7.7 ± 0.6	3.27 ± 0.25
A1503G/IIS4N[a]	−19.8 ± 0.7 (n = 6)	7.5 ± 0.7	3.36 ± 0.31
**IIIS4 neutralisations**			
IIIS4N	No detectable current		
K1025Q/R1028Q/R1031Q/R1034Q/R1037 (IIIS4N+R1041)	No detectable current		
K1025Q/R1028Q/R1031Q/R1034Q (IIIS4N+R1041+R1037)	−19.7 ± 0.9 (n = 5)	8.0 ± 0.5	3.15 ± 0.20
R1034Q	−23.4 ± 0.6 (n = 5)	6.5 ± 0.4	3.82 ± 0.23
A780T/K1025Q/R1028Q/R1031Q/R1034Q	−43.6 ± 0.6	9.3 ± 0.4	2.69 ± 0.12
A780T/K1025Q/R1028Q	−34.7 ± 0.5 (n = 7)	7.7 ± 0.3	3.29 ± 0.11
A780T/R1034Q	−33.5 ± 0.6 (n = 4)	6.1 ± 0.5	4.14 ± 0.31
G1193T/K1025Q/R1028Q/R1031Q/R1034Q	−41.8 ± 0.6	8.2 ± 0.6	3.09 ± 0.21
A1503G/R1034Q	−27.4 ± 0.5 (n = 4)	7.1 ± 0.3	3.56 ± 0.13
**IVS4 neutralisations**			
IVS4N	No detectable current		
R1359Q/R1362Q/R1365Q/K1368Q (IVS4N+R1372)	No detectable current		
R1359Q/R1362Q/R1365Q	No detectable current		
R1359Q/R1362Q	No detectable current		
R1359Q	−6.0 ± 1.0 (n = 5)	5.0 ± 0.6	5.04 ± 0.60
R1365Q	−5.8 ± 1.0 (n = 5)	5.6 ± 1.0	4.47 ± 0.79
G1193T/K1025Q/R1028Q	−35.8 ± 0.7 (n = 7)	8.7 ± 0.6	2.89 ± 0.20

[a]: Data from Beyl et al. https://pubmed.ncbi.nlm.nih.gov/22941337/
[b]: Data from Depil et al. https://pubmed.ncbi.nlm.nih.gov/21685391/
Reference: https://www.ncbi.nlm.nih.gov/pmc/articles/PMC5138263/

S6 Segments: Pore mutations affect voltage-dependent gating of CaV1.2

Mutant	*Vact, mV*	*k, mV*[a]	ΔG, kcal/Mol	*Imax, pA/pF*
Wild-type	−18.6 ± 0.9 (n = 8)	5.9 ± 0.6		15.1 ± 2.8
**IS6 segment**				
L434A	−39.3 ± 0.9 (n = 5)	5.9 ± 0.8	−2.03 ± 0.57	13.3 ± 2.5
L434G	−32.3 ± 0.7 (n = 7)	7.6 ± 0.7	−0.63 ± 0.31	15.7 ± 2.8
L434N	−22.0 ± 0.8 (n = 6)	6.5 ± 0.7	−0.11 ± 0.30	12.3 ± 2.1
L434T	−30.8 ± 1.3 (n = 6)	6.9 ± 0.9	−0.76 ± 0.41	17.1 ± 3.0
L434V	−32.6 ± 0.8 (n = 5)	5.9 ± 0.7	−1.37 ± 0.44	12.6 ± 2.7
L434M	−23.0 ± 1.2 (n = 6)	4.6 ± 0.7	−1.08 ± 0.51	14.3 ± 3.2
L434S	−28.9 ± 0.7 (n = 6)	5.0 ± 0.5	−1.55 ± 0.42	13.7 ± 2.2
L434W	No current	N/A	N/A	N/A
L434P[b]	No current	N/A	N/A	N/A
**IIIS6 segment**				
F1191T	−25.7 ± 1.0 (n = 6)	7.1 ± 0.8	−0.26 ± 0.32	9.6 ± 2.5
V1192T	−16.4 ± 0.9 (n = 7)	6.9 ± 0.9	0.45 ± 0.28	9.1 ± 2.2
G1193T	−49.8 ± 1.0 (n = 7)	5.1 ± 0.6	−3.89 ± 0.72	17.5 ± 3.2
G1193V	−38.1 ± 0.7 (n = 8)	5.6 ± 0.6	−2.14 ± 0.48	12.3 ± 2.7
G1193N	−46.5 ± 1.0 (n = 5)	5.6 ± 0.8	−2.99 ± 0.73	11.2 ± 2.9
G1193M	−33.6 ± 0.7 (n = 8)	6.4 ± 0.6	−1.22 ± 0.36	8.4 ± 2.0
G1193A	−12.1 ± 1.0 (n = 6)	5.7 ± 0.7	0.60 ± 0.28	9.3 ± 1.8
G1193Q	−39.2 ± 0.8 (n = 5)	7.0 ± 0.7	−1.42 ± 0.39	9.8 ± 2.1
G1193P	−37.7 ± 0.7 (n = 7)	6.3 ± 0.6	−1.63 ± 0.39	11.3 ± 2.7
G1193W	No current	N/A	N/A	N/A
F1194T	−16.5 ± 0.9 (n = 7)	7.8 ± 0.9	0.61 ± 0.26	17.3 ± 3.2
V1195T	−28.9 ± 0.7 (n = 7)	5.1 ± 0.7	−1.48 ± 0.51	18.7 ± 3.4
I1196T	−26.6 ± 0.6 (n = 9)	4.9 ± 0.6	−1.31 ± 0.44	18.4 ± 2.7

[a]: in comparison to Wild-Type the changes of slope factors for mutated channels are statistically not significant ($p > 0.05$)
[b]: Data from Kudrnac et al. (https://pubmed.ncbi.nlm.nih.gov/19265197/)
Reference: https://www.ncbi.nlm.nih.gov/pmc/articles/PMC3016219/

S6 Segments: Coupled and Independent Contributions of Residues in IS6 and IIS6 to Activation Gating of CaV1.2

Mutant	*V0.5, act*	*kact*	*V0.5, inact*	*kinact*	*r300*
	mV	*mV*	*mV*	*mV*	*%*
Wild-type	–9.9 ± 1.1 (n = 8)	6.3 ± 0.7	–38.7 ± 1.0 (n = 3)	8.1 ± 0.9	65 ± 4
**IS6 segment**					
L429T	–16.5 ± 0.6 (n = 5)	7.5 ± 0.6	–42.5 ± 1.1 (n = 3)	7.9 ± 0.9	69 ± 6
L434T	–21.9 ± 1.3 (n = 5)	6.9 ± 0.9	–51.1 ± 1.1 (n = 3)	8.6 ± 1.0	63 ± 5
S435T	–11.3 ± 0.7 (n = 5)	6.1 ± 0.6	–40.1 ± 0.9 (n = 3)	7.7 ± 0.8	64 ± 6
S435A	–15.9 ± 0.6 (n = 6)	6.9 ± 0.5	–44.1 ± 0.9 (n = 4)	7.8 ± 0.9	70 ± 5
S435P	–35.8 ± 0.6 (n = 6)	8.8 ± 0.6	–65.7 ± 0.7 (n = 4)	7.3 ± 0.7	5 ± 4[a]
**IIS6 segment**					
L779T	–18.6 ± 0.7 (n = 5)	6.4 ± 0.6	–50.5 ± 1.2 (n = 3)	7.1 ± 0.6	12 ± 5[a]
I781T	–37.7 ± 1.2 (n = 7)	7.2 ± 1.0	–57.8 ± 0.7 (n = 3)	8.9 ± 0.5	47 ± 3[a]
I781P	–47.2 ± 1.1 (n = 8)	6.3 ± 0.6	–68.7 ± 0.8 (n = 5)	5.8 ± 0.5	53 ± 6
**Double mutants**					
L429T/I781T	–44.0 ± 1.0 (n = 7)	8.3 ± 0.9	–61.2 ± 1.0 (n = 4)	8.7 ± 0.9	41 ± 5[a]

L429T/L779T	−22.5 ± 0.8 (n = 5)	6.4 ± 0.7	−49.6 ± 1.3 (n = 3)	8.0 ± 0.7	12 ± 6[a]
L434T/I781T	−50.3 ± 0.8 (n = 5)	5.4 ± 0.8	−70.0 ± 1.6 (n = 3)	8.5 ± 1.2	15 ± 4[a]
L434T/L779T	−32.3 ± 0.8 (n = 7)	7.1 ± 0.7	−58.2 ± 0.8 (n = 5)	8.2 ± 0.9	52 ± 5
S435A/I781T	−35.3 ± 0.9 (n = 6)	8.7 ± 0.7	−59.1 ± 0.9 (n = 4)	8.1 ± 0.8	48 ± 4[a]
S435P/I781T	−43.7 ± 0.9 (n = 5)	9.1 ± 0.9	−64.1 ± 1.1 (n = 3)	8.6 ± 1.0	44 ± 5[a]
S435P/I781P	No current	N/A	N/A	N/A	N/A

[a]: Data are statistically significantly different from wild type ($p < 0.05$). Data for wild type, I781T, and I781P are from Hohaus et al. (12). Mutations G422P, S423P, F424P, N428P, L429P, V430T/V430P, L431T/L431P, G432T/G432P, V433T/V433P, L434P, E437P, F438P, and S439P were not functional

Reference: https://pubmed.ncbi.nlm.nih.gov/19265197/

S6 Segments: Timothy Mutation Disrupts the Link between Activation and Inactivation in CaV1.2 Protein

Construct	*V0.5*	*kact*	V0.5,inact	*kinact*	r3000
	mV	*mV*	*mV*	*mV*	
**Wild-type**					
	−18.4 ± 0.8 (n = 9)	6.0 ± 0.7	−41.5 ± 0.9 (n = 5)	6.4 ± 0.9	0.35 ± 0.03
**IS6 G432a**					
G432A	−14.2 ± 0.6 (n = 7)	6.2 ± 0.5			0.77 ± 0.05
G432M	−23.2 ± 0.6 (n = 7)	7.7 ± 0.6			0.68 ± 0.07
G432N	−34.4 ± 0.4 (n = 7)	6.5 ± 0.5			0.72 ± 0.04
G432S	−19.2 ± 0.7 (n = 7)	5.9 ± 0.7			0.74 ± 0.07
G432V	−12.0 ± 0.6 (n = 6)	5.7 ± 0.5			0.86 ± 0.04
G432W	−33.7 ± 0.6 (n = 8)	4.7 ± 0.4			0.58 ± 0.04
**IIS6 A780**					
A780G	−29.4 ± 0.6 (n = 5)	6.2 ± 0.6	−54.4 ± 1.3 (n = 4)	6.2 ± 1.1	0.11 ± 0.06
A780N	−43.8 ± 1.0 (n = 7)	6.8 ± 0.9	−67.4 ± 0.7 (n = 4)	4.6 ± 0.9	0.25 ± 0.04
A780P	−45.0 ± 0.7 (n = 5)	5.9 ± 0.8	−39.4 ± 1.2 (n = 4)	5.2 ± 1.9	0.34 ± 0.07
A780T	−44.0 ± 0.6 (n = 5)	5.1 ± 0.6	−59.8 ± 0.9 (n = 4)	7.5 ± 0.9	0.24 ± 0.04
A780V	−34.6 ± 0.8 (n = 8)	6.6 ± 0.7	−54.3 ± 0.8 (n = 4)	4.8 ± 0.8	0.10 ± 0.05
A780W	−49.3 ± 0.6 (n = 7)	5.9 ± 0.5	−68.0 ± 0.8 (n = 4)	6.3 ± 0.9	0.16 ± 0.05
**IIIS6 G1193b**					
G1193A	−12.1 ± 1.0 (n = 6)	5.7 ± 0.7	−25.2 ± 1.1 (n = 6)	8.8 ± 0.9	0.27 ± 0.07
G1193M	−33.6 ± 0.7 (n = 8)	6.4 ± 0.6	−52.8 ± 1.0 (n = 4)	6.1 ± 0.9	0.34 ± 0.08
G1193N	−46.5 ± 1.0 (n = 5)	5.6 ± 0.8	−61.3 ± 0.9 (n = 4)	6.9 ± 0.9	0.38 ± 0.08
G1193P	−37.7 ± 0.7 (n = 7)	6.3 ± 0.6	−64.6 ± 1.0 (n = 4)	6.1 ± 0.8	0.18 ± 0.07
G1193Q	−39.2 ± 0.8 (n = 5)	7.0 ± 0.7	−64.4 ± 1.2 (n = 4)	8.4 ± 1.1	0.29 ± 0.08
G1193T	−49.8 ± 1.0 (n = 7)	5.1 ± 0.6	−70.0 ± 1.1 (n = 5)	5.4 ± 1.1	0.16 ± 0.07
G1193V	−38.1 ± 0.7 (n = 8)	5.6 ± 0.6	−60.4 ± 0.8 (n = 5)	7.5 ± 0.6	0.14 ± 0.08
**IVS6 A1503**					
A1503G	−41.4 ± 0.8 (n = 5)	5.8 ± 0.5	−54.0 ± 1.0 (n = 4)	4.9 ± 0.8	0.31 ± 0.07
A1503M	−29.2 ± 0.7 (n = 8)	5.9 ± 0.7	−60.1 ± 1.0 (n = 4)	5.5 ± 1.0	0.26 ± 0.08
A1503N	−31.5 ± 0.7 (n = 5)	6.3 ± 0.6	−45.1 ± 1.1 (n = 4)	6.0 ± 1.0	0.32 ± 0.09
A1503T	−17.9 ± 0.8 (n = 7)	8.7 ± 0.8	−38.0 ± 1.2 (n = 4)	6.9 ± 1.1	0.29 ± 0.09
A1503V	−11.4 ± 0.8 (n = 5)	7.2 ± 0.6	−37.2 ± 1.2 (n = 4)	6.8 ± 1.2	0.24 ± 0.06
A1503W	−16.9 ± 0.6 (n = 9)	6.6 ± 0.6	−38.9 ± 1.3 (n = 4)	7.5 ± 0.8	0.33 ± 0.09
A1503P	No current	N/A	N/A	N/A	N/A
**Double mutation**					

G432S/S435G	−19.8 ± 0.9 (n = 5)	6.3 ± 0.7	−39.2 ± 1.2 (n = 4)	5.7 ± 1.1	0.54 ± 0.07

Reference: https://www.ncbi.nlm.nih.gov/pmc/articles/PMC3173108/
