# Supplementary material for: Permeabilizing Cell Membranes with Electric Fields
Source: Cancers (Basel). 2021 May 10;13(9):2283. doi: 10.3390/cancers13092283 (PMC8126200; doi:10.3390/cancers13092283)
Supplement: Supplementary file 1 [file cancers-13-02283-s001.zip › cancers-1169693-supplemental-tables/20210314 Table S2_final.pdf]

SUPPLEMENTARY TABLE S2. Parameters of bioelectrorheological model

Parameter	Description	Bioelectrorheological Model		Electroporation Model	TTFields	Notes
		Membrane Destabalization (Contributes to Electroporation)	Electrofusion			
***Frequency of Electric Field**	At high frequencies shear stress within the membrane decreases and reaches a constant value corresponding to dielectric stress solely	Yes, f = 7 x 10^1 Hz to 3 x10^5 Hz		Yes	Yes 200 kHz	*Ratio of cells susceptible to electroporation: cells susceptible to electrodestruction constant at low frequencies but decreased at higher frequencies (f ≈ 1.0x10^4 Hz), implying both processes are due to different mechanisms and/or involve different molecular structures.
**Initial outer cell radius**	-	Yes				
**Reversibility**	Under the applied stress only reversible, nondamaging viscoelastic deformations were developed	Yes		Yes	Yes	
**Susceptibility to electroporation**	Expression of extensil stress leading to electroporation, increased with frequency, highest at f ≈ 10^5 Hz	Yes				
**Induced cell membrane potential**	Increased with frequency	Yes				
**Susceptibility to destabalization**	Dependent on frequency, increases with frequency, varies depending on cell type, f = 10^4 Hz a steep increase in this susceptibility occurred possibly explianed by resonance oscillations of the susceptible structures and from accompanying increase in heat supplied to the system	Yes, highest at f = 10^5 Hz		Yes		
**Applied extensil stress**	Dependent on frequency, increases with frequency, varies depending on cell type, f = 10^4 Hz a steep increase in this susceptibility occurred possibly explianed by resonance oscillations of the susceptible structures and from accompanying increase in heat supplied to the system	Yes				
**Width of the pulse of the alternating field**	200 ms for myeloma and 2 s for the N. crassa cells lead to electrodestruction	Yes				
**Voltage amplitude**	Voltage amplitude at which electrodesturciton begins to be observed	Yes				
**Field strength**	Of electric field	Yes				
**Coefficient of combination α**	Describes the unknown part of heat essential in destabilization processes.	Yes				
**Viscocity of cellular structures (coefficient of viscocity)**	Influenced by membrane proteins and cytoskeletal network. Sigmoidal decrease in the viscosity of structures reactive to the frequency of stress oscillations at f = 10^4 Hz to 10^5 Hz, also affects membrane susceptibility to destabalization	Yes				
**Heat energy**	Heat energy was mainly responsible for destabilization of the membrane at high frequencies, Joule heat amplified at pore openings, influencing membrane susceptibility	Yes	Yes			
**Ionic strength of the external medium**	Influence the mechanical susceptibility of Tib9 cells to electroporation	Yes		Yes		
**Energy of intermolecular interactions responsible for the integrity of the membrane.**	Proportional to yield of electrofusion, thermal chaotic motion may promote mixing of membrane molecules of two neighboring cells remaining at a short distance, affecting electrofusion		Yes			
**Density of energy expended**	Higher for electrodestruction than electroporation, increases for electroporation with increase of external medium conductivity.	Yes		Yes		
**Membrane inertia**	Related to membrane viscocity	Yes	Yes			
**Cytoplasm and cytoskeletal interaction**	Influence membrane viscocity and intertia	Yes				
**Initial tension in membrane**	In absence of electric field	Yes		Yes		
**Density of isothermal work performed against the intermolecular forces and of intrinsic heat**	Related to disruptive effects in the membrane and increase in the energy of intermolecular interactions responsible for the integrity of the membrane	Yes	Yes	Yes		
**Membrane viscocity**	-	Yes	Insignifcant effect			
**Complex dielectric permeability**	-	Yes				
**Complex specific electric conductivity**	-	Yes				
**Mechanical stress**	Theoretically evaluated by including into calculations the dielectric permittivity and electric conductivity of the external and internal medium and the membrane, nonzero magnitude of field frequency, variations of cytoplasmic pressure (bound to cell deformation), and curvature of cell surface.	Yes				
**Length of deformed cell along the electric field direction**	-	Yes				
**Cell membrane thickness**	-	Yes				
**Solid angle element**	-	Yes				
**Electrode radius**	-	Yes				
**Distance between electrodes**	-	Yes				
**Dielectric permeability**	-	Yes				
**Dielectric permeability of vacuum**	-	Yes				
**Surface pressure**	-	Yes				
**Complex amplitude of extensil stress oscillations**	-	Yes				

References	Notes
https://pubmed.ncbi.nlm.nih.gov/2601349/	
https://pubmed.ncbi.nlm.nih.gov/2533955/	Rows 3-7 of this supplemental table are mentioned in Table 3 of main manuscript. Rows 8-35 are additional.
https://pubmed.ncbi.nlm.nih.gov/1387010/	

https://pubmed.ncbi.nlm.nih.gov/8789120/	
https://pubmed.ncbi.nlm.nih.gov/8369458/	
