# Supplementary material for: Permeabilizing Cell Membranes with Electric Fields
Source: Cancers (Basel). 2021 May 10;13(9):2283. doi: 10.3390/cancers13092283 (PMC8126200; doi:10.3390/cancers13092283)
Supplement: Supplementary file 1 [file cancers-13-02283-s001.zip › cancers-1169693-supplemental-tables/20210314 Table S3_final.pdf]

SUPPLEMENTARY TABLE S3. Parameters of electroporation model

Parameter	Description	Bioelectrorheological Model	Electroporation Model			TTFields	Other Information	References
			Direct Current (DC) Field/Rectangular Pulses	Radio Frequency (RF) Field	Alternating Current (AC) Field			
**Time of application**	Duration that the electric field applied		Short: 10μs (rectangle pulse) or exponential decay by discharging capacitor	Medium: 2ms	Long: 40ms	Continuous and long: Ideally for up to hours at a time		https://pubmed.ncbi.nlm.nih.gov/30534421/<br>https://pubmed.ncbi.nlm.nih.gov/29260225/<br>https://pubmed.ncbi.nlm.nih.gov/2819230/<br>https://www.btxonline.com/media/wysiwyg/protocol_db/Electroporation_Optimization_Guide.pdf.
**Optimal frequencies**	Frequency at which the AEF model is most effective	Susceptibility to electroporation varies nonlinearly with frequency	No: DC frequency is 0 kHz	Yes: RF frequency is 40 kHz (from 20 kHz to 300 GHz)	Yes: 10 kHz to 1 MHz, optimal at 40-120 kHz	Yes: 200 kHz	*DNA transfection observed to be most effective in the 100-1000 Hz range	https://pubmed.ncbi.nlm.nih.gov/30534421/<br>https://pubmed.ncbi.nlm.nih.gov/8369458/<br>https://pubmed.ncbi.nlm.nih.gov/2819230/
**Intensity/field strength**	Relative level of electric field intensity delivered		Yes: 0.25-3 kV/cm	Yes: optimal at E=1.8-2.1 kV/cm (but oscillating)	Yes: experimental values of 500-700 V/cm	Yes: 2 V/cm	*Strength of the electric field is proporational to the transmembrane voltage (Kotnik et al.)	https://pubmed.ncbi.nlm.nih.gov/2819230/<br>https://pubmed.ncbi.nlm.nih.gov/30534421/
**Pore formation**	Pore formation as a result of membrane destabilization	Yes	Yes: 1-ms 40 kV/m pulse: ~341,000 pores, of which 97.8% are small (≈1 nm radius) and the avg radius of large pores is 22.8 ± 18.7 nm, although some pores grow to 419 nm, diameter of pores ranges from 25-120nm	Yes		Yes: increased number of holes >51nm^2 (radius of 4.1nm) in size, avg size of holes 240.6 +/- 91.7nm^2	*And ion channel activation by short (submicrosecond) pulses with high amplitudes	https://pubmed.ncbi.nlm.nih.gov/17056739/<br>https://pubmed.ncbi.nlm.nih.gov/2819230/<br>https://pubmed.ncbi.nlm.nih.gov/30534421/<br>https://pubmed.ncbi.nlm.nih.gov/8369458/
**Reversibility**	Are the effects temporal or permanent?	Yes, formation of pores and membrane damage are temporal	Yes and No: up to a critical potential (depends on time and field strength), pore closing is fast (ms to min)	Yes	Unknown	Yes	*Irreversible electroporation has been studied for cancer treatment	https://pubmed.ncbi.nlm.nih.gov/30534421/<br>https://pubmed.ncbi.nlm.nih.gov/1387010/<br>https://pubmed.ncbi.nlm.nih.gov/30669316/<br>https://pubmed.ncbi.nlm.nih.gov/30786231/
**Maximal membrane potential**	Equal to or greater than the potential at which the membrane is perforated at the 2 loci facing the electrodes		$V_m = 1.5rEcos\theta$		$\Delta\psi_{membr} = \frac{1.5aE\cos\theta}{\left[1+(\omega\tau)^2\right]^{1/2}}$	$\Delta V = f_s E_e R \cos\theta \frac{1}{1+j\omega\tau_m}$	*at low frequencies the intensity (E) is independent of the ac frequency. At high frequencies, E increases with frequency (Marszalek et al.) However, this is disputed in Zhan et al. which shows that frequency does matter at lower ranges and points out that frequency is inversely related to transmembrane potential	https://pubmed.ncbi.nlm.nih.gov/2248989/<br>https://pubmed.ncbi.nlm.nih.gov/2819230/<br>https://pubmed.ncbi.nlm.nih.gov/22516092/<br>https://pubmed.ncbi.nlm.nih.gov/31940516/
**Membrane destabilization**	Is the plasma membrane affected by the AEF model?	Yes: shear stress leads to physical deformations of cell shape and extensil stress leads to membrane destabalization and electroporation	Yes: electrical breakdown of membrane and surface tension	Yes	Yes: electrical breakdown and dielectrophoresis	Yes		https://pubmed.ncbi.nlm.nih.gov/30786231/<br>https://pubmed.ncbi.nlm.nih.gov/2819230/<br>https://pubmed.ncbi.nlm.nih.gov/30534421/
**Effect on ion channels**	Findings that ion channels can be affected by electric fields		Yes: ion channel conductivity increased by short (submicrosecond) pulses with high amplitudes, however, dependent on a variety of factors			Yes: CaV1.2 activation		https://pubmed.ncbi.nlm.nih.gov/21080060/<br>https://pubmed.ncbi.nlm.nih.gov/30669316/
**Effect on cytoskeleton**	Findings show that electric fields can affect the polar subunits such as tubulin, actin, septin, etc		Yes: F-actin and beta-tubulin disprupted—and this effect is reversible (however unconfirmed whether this is a direct or indirect result of the electric field exposure)			Yes: affects tubulin and septin		https://pubmed.ncbi.nlm.nih.gov/17172418/<br>https://pubmed.ncbi.nlm.nih.gov/26658786/
**Cell survival**	Survival of the exposed cell	Yes	Yes: depends on intensity and time	Yes: at optimal intensity survivability is ~80-70% (RF ~3x more gentle than DC field)	Yes			https://pubmed.ncbi.nlm.nih.gov/2819230/<br>https://pubmed.ncbi.nlm.nih.gov/1387010/
**Impact on cancer cells**	Has the model been studied on cancer cells?	Not studied	Yes: study on calcium electroporation found that cancer cells were much more sensitive to electroporation than normal cells		Yes: myeloma cells found to have a lower critical membrane potential (point of membrane perforation) 0.95 V versus 1 V in normal cells	Yes	*Studies suggest that differences could be caused by a difference in repair capacities	https://pubmed.ncbi.nlm.nih.gov/2248989/<br>https://pubmed.ncbi.nlm.nih.gov/28681243/<br>https://pubmed.ncbi.nlm.nih.gov/31991784/
**Electric field exposure**	What duration/type of electric field	Yes: oscillating	Yes: pulsing	Yes: oscillating	Yes: alternating (aka oscillating)	Yes: alternating (aka oscillating)	*Oscillating electric fields may cause increased mechanical stress on the membrane compared to pulsing (Chang et al.).	https://pubmed.ncbi.nlm.nih.gov/2819230/<br>https://pubmed.ncbi.nlm.nih.gov/2601349/<br>https://pubmed.ncbi.nlm.nih.gov/30534421/
**Localization**	The effects of the electric field are targeted and localized in a predicted area		Yes	Yes	Yes	Yes		https://pubmed.ncbi.nlm.nih.gov/30786231/<br>https://pubmed.ncbi.nlm.nih.gov/30786231/
**Probability of poration**	How effective is the AEF model at porating the membrane?	Susceptibility to electroporation (s[p]) was represented by the reciprocal of the extensil stress needed for electroporation (σ0e[p]), i.e., s[p] = (σ0e[p])-1; increases with frequency and decreases with increasing external medium conductivity.		Transfection rate for RF is 2x greater than for DC	At the 2 loci it is equal for AC and DC fields			https://pubmed.ncbi.nlm.nih.gov/2819230/<br>https://pubmed.ncbi.nlm.nih.gov/8369458/
**Membrane electrical conductivity**	Any changes in electrical conductivity/voltage?		Yes: increases within 1μs after onset of electrical pulse					https://pubmed.ncbi.nlm.nih.gov/30786231/
**Cell diameter**	Generally smaller cell diameters require higher voltage and larger diameters require lower voltages		Yes	Yes	Yes			https://www.btxonline.com/media/wysiwyg/protocol_db/Electroporation_Optimization_Guide.pdf
**DNA concentration**	Increase in DNA concentration directly increases transfection efficiency		Yes	Yes	Yes			https://pubmed.ncbi.nlm.nih.gov/10932162/<br>https://www.btxonline.com/media/wysiwyg/protocol_db/Electroporation_Optimization_Guide.pdf
**Temperature**	The temperature affects the efficiency of electroporation		Yes: ideal temp. still being investigated but heating at around 42ºC has improved transfection efficiency, while keeping at lower temp delays pore closure	Yes	Yes			https://pubmed.ncbi.nlm.nih.gov/28924200/<br>https://www.btxonline.com/media/wysiwyg/protocol_db/Electroporation_Optimization_Guide.pdf
**Electroporation buffer**	Ionic strength, pH and volume of liquid have an effect on resistance which in turn affects pulse length or time constant		Yes	Yes	Yes			https://www.btxonline.com/media/wysiwyg/protocol_db/Electroporation_Optimization_Guide.pdf
**Cuvette gap size**	The distance between electrodes has an optimal gap size and can influence optimal field strength		Yes: optimal size for mammalian cells is 2-4mm	Yes	Yes	No	*field strength = voltage/gap size	https://www.ncbi.nlm.nih.gov/pmc/articles/PMC4642621/<br>https://www.btxonline.com/media/wysiwyg/protocol_db/Electroporation_Optimization_Guide.pdf
**Number of pulses**	Number of pulses typically increases as voltage decreases to progressively permeate the membrane		Yes			No: not necessarily number of pulses but yes frequency		https://www.btxonline.com/media/wysiwyg/protocol_db/Electroporation_Optimization_Guide.pdf
**pH**	Electroporation efficiency and cell survivability impacted by extracellular pH		Yes: permeabilization threshold constant at pH 7.8 and 6.5, after exposure to pulse amplitude greater than threshold cell survivability greater at 6.5pH but efficiency decreased					https://pubmed.ncbi.nlm.nih.gov/31404809/

**Cell damage**	Side effects of the electrical field that lead to cell damage	Yes: extensil stress destabalizes cell membrane and leads to poration, shear stress leads to physical defomrations of cell	Yes: electrolysis and joule heating at site of electrodes			No			https://pubmed.ncbi.nlm.nih.gov/30804395/ https://pubmed.ncbi.nlm.nih.gov/29681995/ https://pubmed.ncbi.nlm.nih.gov/1387010/ https://pubmed.ncbi.nlm.nih.gov/8789120/

Notes:

Parameters in rows 3-13 were used to create Table 4 in the main manuscript. Rows 14-25 are additional parameters.
