# Supplementary material for: Permeabilizing Cell Membranes with Electric Fields
Source: Cancers (Basel). 2021 May 10;13(9):2283. doi: 10.3390/cancers13092283 (PMC8126200; doi:10.3390/cancers13092283)
Supplement: Supplementary file 1 [file cancers-13-02283-s001.zip › cancers-1169693-supplemental-tables/20210314 Table S4_final.pdf]

SUPPLEMENTARY TABLE S4. Differences in resting membrane potential (Vm) between cancer and non-cancer cells

Brain Cancer Membrane Parameters*					Electrophysical properties of neurons**	Electrophysiology of brain cells***	
Parameter	Glioma Cell Lines		Immature Astrocytes	Mature Astrocytes	Avg of different types of neurons in the motor cortex of the cat	Pooled data from 82 neurons	Pooled data from 69 myenteric glial cells
	D54	STTG1					
Resting membrane potentials (mV)	-38.5 ± 4.2	-28.1 ± 3.5	-51.1 ± 3.7	-75.2 ± 0.56	-63 to -66	−46 ± 13	−55 ± 10 mV
Input Resistances (MΩ)	260.6 ± 64.7	687.2 ± 160.3	627.5 ± 164	25.4 ± 7.4	8.3 to 19.8	392 ± 203	90 ± 42
Barium (Ba2+) sensitive Kir currents (nS/pF)	0.169 ± 0.033	0.244 ± 0.04	0.474 ± 0.12	4.06 ± 1.1			
Action potential amplitudes (mV)					72 to 77		
Firing Threshold (mV)					-54		
Membrane time constant (ms)					7.2 to 15.1		

*Whole-cell voltage-clamp recordings were obtained by standard methods. Reference:https://pubmed.ncbi.nlm.nih.gov/14999814/

**Values measured with intracellular voltage recordings and single-electrode voltage-clamp (SEVC) techniques. Reference: https://pubmed.ncbi.nlm.nih.gov/8350127/

***Whole-cell voltage clamp recordings were made using an Axopatch 200A amplifier. Reference: https://pubmed.ncbi.nlm.nih.gov/10762619/

Mutational status,* cell radius and dielectric properties of 5 GBM lines studied here.

Cell line	p53	PTEN	Radius ± SE μm	C m ± SE μF/cm2	C C ± SE pF	Folding φ	N, cell number	a ± SE μm	β ± SE	P w ± SE μm/s	α ± SE nmol/(s·cm^2)	N, cell number
DK-MG	wild type	wild type	6.3±0.1	1.88±0.07	9.4±0.2	2.38	420	8.1±0.2	0.73±0.01	0.59±0.10	0.25±0.01	40
GaMG	mutated	wild type	8.5±0.1	3.17±0.12	28.8±0.9	4	300	10.0±0.3	0.61±0.05	4.17±0.07	0.46±0.01	30
U87-MG	wild type	mutated	6.7±0.1	2.82±0.09	15.9±0.3	3.5	420	7.6±0.2	0.71±0.02	3.02±0.15	0.37±0.01	50
U373-MG	mutated	mutated	7.1±0.1	4.00±0.12	25.3±0.7	5.25	360	8.8±0.3	0.64±0.08	4.05±0.63	0.35±0.02	20
SNB19	mutated	mutated	6.8±0.1	3.71±0.15	21.6±0.7	4.63	360	8.5±0.4	0.68±0.07	2.21±0.16	0.48±0.02	30

Reference: https://pubmed.ncbi.nlm.nih.gov/24498019/
